# Supplementary material for: PESSA: A web tool for pathway enrichment score-based survival analysis in cancer
Source: PLoS Comput Biol. 2024 May 8;20(5):e1012024. doi: 10.1371/journal.pcbi.1012024 (PMC11078417; doi:10.1371/journal.pcbi.1012024)
Supplement: Attachment — Submitted filename: Response Letter.docx [file pcbi.1012024.s006.docx]

**Manuscript Number:** PCOMPBIOL-D-23-01604

**Title:** PESSA: A Web Tool for Pathway Enrichment Score-Based Survival Analysis in Cancer

**Journal:** PLOS Computational Biology

**Author(s):** Hong Yang, Ying Shi, Anqi Lin, Chang Qi, Zaoqu Liu, Quan Cheng, Kai Miao, Jian Zhang, Peng Luo

Dear Editor:

On behalf of my co-authors, I am resubmitting the attached manuscript, titled ***‘PESSA: A Shiny App for Pathway Enrichment Score-Based Survival Analysis in Cancer’ (Ms. Ref. No.: PCOMPBIOL-D-23-01604)***, for reconsideration for publication in ***PLOS Computational Biology***. We are very thankful to you and the reviewers who have devoted valuable time and effort to reviewing our manuscript. The reviewers’ comments are highly insightful and have greatly helped us improve our manuscript's quality. We have carefully reviewed the comments and have revised the manuscript accordingly. We submitted 3 files, including a revised manuscript, a track changes file (to highlight differences between the revised and the original manuscript), and the response letter. Our responses are given in a point-by-point manner below. Changes to the manuscript are shown in blue.

We should also add that there will be a subsequent version of PESSA for local deployment, which has not yet been deployed.

We hope that these revisions are sufficient to make our manuscript suitable for publication in the Genes & Diseases and look forward to hearing from you at your earliest convenience.

Yours sincerely,

Corresponding author:

Dr. Quan Cheng

Department of Neurosurgery, Xiangya Hospital, Central South University, Changsha, Hunan, China.

National Clinical Research Center for Geriatric Disorders, Xiangya Hospital, Central South University, Changsha, China.

Email: chengquan@csu.edu.cn

Dr. Kai Miao

Cancer Centre and Institute of Translational Medicine, Faculty of Health Sciences, University of Macau, Macau SAR, 999078 China.

MoE Frontiers Science Center for Precision Oncology, University of Macau, Macau SAR, 999078 China.

Email: kaimiao@um.edu.mo

Dr. Jian Zhang

Department of Oncology, Zhujiang Hospital, Southern Medical University, Guangzhou, Guangdong, China.

Tel: 86-13925091863

Fax: 020-61643888

Email: [zhangjian@i.smu.edu.cn](mailto:zhangjian@i.smu.edu.cn)

Dr. Peng Luo

Department of Oncology, Zhujiang Hospital, Southern Medical University, Guangzhou, Guangdong, China.

Tel: 86-18588447321

Fax: 020-61643888

Email: [luopeng@smu.edu.cn](mailto:luopeng@smu.edu.cn)

**Response to Reviewer 1:**

Reviewer #1:  Yang et al. presented PESSA, a shiny app for correlating pathway enrichment scores with survival outcomes. The team has calculated ssGSEA scores for the entire TCGA and 214 datasets from GEO, which covers 21 cancer types based on gene sets from MsigDB. The Shiny app provides an excellent interface for users to search pathways that might be associated with survival. The tool will be an excellent resource to perform high-level pan-cancer analysis. However, the current analysis neglects the heterogeneity of the patient cohort in each study, specifically related to histology, clinical grade, and treatment context, which can affect the interpretation of the results. There should be more effort to assess these covariates as additive effects and interactions within the systematic analysis via the CoxH model. Overall, the tool is of interest to the scientific community, but I would recommend the author to significantly expand its functionalities.

Response:

Thank you very much for your detailed and useful comments. We have addressed each of them and showed them in a point-by-point manner as follows.

Major Comments.1: The heterogeneity of the patient cohort needs to be included in the current resource. The patients need to be subsetted according to histology, clinical grade, and treatment context. I would also recommend incorporating these features as additive and interacting covariates in the CoxH model.

Response:

Dear reviewers, Thank you for your valuable suggestions. We strongly agree with you that patient heterogeneity may lead to differences in disease response and survival prognosis among individuals. However, because some samples do not contain detailed clinical information such as tumor grade and histological subtype in the GEO and TCGA databases, and because there are differences in the amount of clinical information between datasets, subgroup analyses are very likely to face a limited sample size, thus affecting statistical validity. For this reason, PESSA does not provide online subgroup analysis. As an alternative, we provided users with as much detailed information as possible about the treatment of the sample and provided hyperlinks to detailed pages of the dataset for further exploration. The content you suggest has been added to the limitations section of the article (Page 18-19, lines 365-371), and we will endeavor to break through this limitation in the future.

Major Comments.2: If Cox analysis is used, the author may include additional test statistics, such as the concordance index and chi-square test, to evaluate the proportion hazard assumption.

Response:

Thank you for your valuable suggestion that including additional test statistics in Cox proportional risk model analyses would indeed allow for a more comprehensive assessment of the quality and applicability of the model. Therefore, we added the Schoenfeld residual test to the Cox continuous variable analysis by using the cox.zph() function from the survival package. We add this in the corresponding Methodology section of the manuscript (Page 12, lines 233-236).

Major Comments.3: As the author states, the pathway tools might also be helpful in prioritizing gene features. It might be useful to perform a systematic screen for individual genes.

Response:

Thank you for your valuable suggestions. We likewise agree that correlation studies between single gene expression levels and tumor survival are valuable in deepening the understanding of cancer biological mechanisms, discovering biomarkers, and improving prognostic assessment. Another web tool developed by our team, PanCanSurvPlot, precisely employs single-gene level searches to discuss the relationship between single genes and tumor survival. Therefore, to avoid functional duplication, PESSA only discusses the relationship between pathway level and tumor survival.PanCanSurvPlot tool homepage (http://www.pancansurvplot.com/) and preprints (https://www.biorxiv.org/content/10.1101 /2022.12.25.521884v1) links are attached.

Major Comments.4: The provided MsigDB gene set is limited, and many of the immune-associated gene sets (also available to MsigDB) were not included. Given the significant interest in immunotherapy, the authors should also include results associated with these gene sets.

Response:

Thank you for your valuable suggestions! A total of 25 pathway datasets related to immune checkpoint inhibitor therapy from EGA, GEO, and supplementary tables of articles have been added. The detailed pathway dataset names and their corresponding genes are provided in Supplementary table 3, and the methodology and results sections of the article have been supplemented accordingly (Page 10, line 184-187; Page 13, line 256-258).

Major Comments.5: Since the current resource is designed for pan-cancer analysis, there should be an additional summary of pathways relevant in each histology, such as a) pathways implicated across squamous malignancies, b) pathways implicated in Herpes virus, c) pathways implicated in samples derived from metastatic events, d) pathways implicated in samples from pre or post immunotherapy treatment. There should be existing literature on the four studies, and it would be nice to provide examples in which PESSA was able to confirm or reject existing studies.

Response:

Thank you very much for such detailed and enlightening suggestions. In response, we added to our manuscript a validation of the predictive effect of the activation status of immunotherapy-associated pathways on the prognosis of patients receiving immunotherapy (Page 16, lines 301-307). We analyzed patients with urothelial metastatic epithelial carcinoma (BLCA) treated with PD1 inhibitors using HALLMARK - DNA REPAIR as a target.PESSA results demonstrated that patients with BLCA treated with PD1 inhibitors who exhibited high activation of the DDR pathway had significantly longer the overall survival (OS) time. Similarly, high activation of the DDR pathway in melanoma patients treated with PD1 inhibitors was also associated with a better prognosis. These results are consistent with previous findings.

Minor comments.1: The download feature is limited to the high-level summary with only 20-100 items that are viewable/downloadable at a time. I would recommend the author add the ability for the users to download the complete table.

Response:

Thank you for your review.PESSA has the ability to download full data. As shown in the picture, by clicking on this button, you can get a csv table of the results of the analysis of all the cancers related to the pathway you are interested in.

Minor comments.2: Endpoints to the survival analysis should be defined, such as OS, PFS, DMFS, DFS, MFS, and BCR.

Response:

Thank you for your review.PESSA provides detailed survival information full name and its abbreviated counterpart in the About page FAQ as shown below.

Reviewer #2: This manuscript describes a simple but useful tool to aid in analyzing gene set activation as a biomarker in cancer for prognosis. The tool is built as an R Shiny app that comes preloaded with many gene sets and expression data from a range of cancers. The app is easy to use and can provide statistical results and figures. Overall, I think the approach does have the potential to be of wide interest to cancer researchers. However, I do have some concerns.

Response:

We would like to thank you for providing your constructive and detailed review comments on our manuscript. Those suggestions have helped us to significantly enhance the quality of the manuscript. We have addressed each comment and showed them in a point-by-point manner as follows.

Minor.1: On line 63, there is a typo "820,2023"

Response:
Thank you for your careful review and for pointing out our mistake. We apologize for the error, it was a mistake in our writing and we did not check it during the review and have corrected it to "609,820". (Page 6, line 103)

Minor.2: On line 146, it is unclear what normalization was applied to arrays (which needs a citation) vs. sequencing data.

Response:

Thank you for your comments. For standardization: we used the normalizeBetweenArrays function of the limma package for standardization, which is used for batch effect removal and between-sample standardization that removes between-group differences. We have added details on standardization in the Methodology section of the article (Page 10, line 187-193).

Minor.3: On line 174, it is not stated how the optimal cut-off is obtained.

Response:

Thank you for your comments. The computation of our optimal cutoff value grouping is based on the sur_cutpoint function of the survminer package [1]which references Mizuno H et al [2]: “Survival analysis in PrognoScan employs the minimum P-value approach to find the cutpoint in continuous gene expression measurement for grouping patients. First, patients are ordered by expression value of a given gene. Next, patients are divided into two (high and low) expression groups at all potential cutpoint, and the risk differences of the two groups are estimated by log-rank test. Then, optimal cutpoint that gives the most pronounced P-value (Pmin) is selected.” In addition, this method is also widely used in bioinformatics analysis and prognostic analysis [3]. In the methodology section of the article we have made additional remarks accordingly to your suggestion (Page 12, line 225-227).

Reference

1.	Alboukadel K, Marcin K, Przemyslaw B. survminer: Drawing Survival Curves using “ggplot2.” [cited 8 Apr 2023]. Available: https://cran.r-project.org/web/packages/survminer/index.html

2.	Mizuno H, Kitada K, Nakai K, Sarai A. PrognoScan: A new database for meta-analysis of the prognostic value of genes. BMC Med Genomics. 2009;2. doi:10.1186/1755-8794-2-18

3.	Nagy Á, Lánczky A, Menyhárt O, Győrffy B. Validation of miRNA prognostic power in hepatocellular carcinoma using expression data of independent datasets. Sci Rep. 2018;8: 9227. doi:10.1038/s41598-018-27521-y

Minor.4: There is no ability to control for confounding in models.

Response:

Thank you for your valuable comments on our article. We understand your considerations related to the control of confounders in survival analysis. However, we do not believe that it is necessary to include confounding variables to control for confounding in a Kaplan-Meier survival analysis, as in a regression analysis, which is a descriptive analytical method used to characterize and compare the distribution of survival times among different groups. This type of analysis does not itself assess the causal relationship between risk factors and outcomes, and therefore does not need to control for confounding variables. In contrast, regression analyses fit the mathematical relationship between survival time and risk factors and therefore require adjustment for confounding variables to minimize confounding bias. In our study, our aim was to characterize and compare the distribution of survival between the two groups of patients, rather than to assess the effects of risk factors. We have also made it clear in the Discussion that causality was not assessed in this study. Thank you very much for raising this issue, which we will add and clarify in the revised manuscript (Page 12, lines 238-240). Thank you again for your valuable comments!

Minor.5: Gene sets are only given by name and there is no description, making them hard to use.

Response:

Thank you for your valuable suggestion, we have updated it in the PESSA webpage. You can get the profile of the selected gene set directly under Plot Tab and can jump to the details page of the pathway dataset via hyperlinks as shown below. We have also added it in Supplementary table5.

Minor.6: There is no ability to upload user data, which limites the utility of the tool.
Response:

Thank you for your suggestion. It is true that the function of uploading data can better meet the needs of users' personalized analysis. However, in order to support users to upload microarray or sequencing expression profiles, we must take into account the necessary pre-processing steps such as expression profiling data quality control, standardization, data cleaning, outlier screening, gene name conversion, and so on. For the shiny web tool, it is extremely difficult to analyze the data quality and ensure the running speed at the same time.

However, we have taken into consideration that users may have the ability to upload customized pathway datasets. We have set up a message board function under the About Tab to encourage and support users to upload interested pathways through the comment box. We will update the tool and notify users by email. We will add and explain this in the revised draft. (Page 14, line 270-271; Page 18, line 361-363)

Major.7: The manuscript assumes that the overall method of using ssGSEA is useful compared to existing methods, of which there are many. However, it is not really established in either the background or the results.

Response:

Thank you for raising this important point. We agree that the usefulness and novelty of using ssGSEA for survival analysis should have been better demonstrated in the manuscript, and we have added accordingly in Discussion. (Page 17, lines 326-332)

ssGSEA has the feature of generating pathway activation scores for each sample, allowing for a more fine-grained, personalized pathway association with survival and thus explaining heterogeneous activation between patients. In addition, ssGSEA scores have been shown to better summarize known biology and validate experimental assays of pathway activity than enrichment assays such as GSEA, GSVA, ORA, etc[4,5]. As well as sequential ssGSEA scores allow for more robust survival modeling using the scores as a predictor variable as compared to dichotomous enrichment calls.

Reference

4.	Barbie DA, Tamayo P, Boehm JS, Kim SY, Moody SE, Dunn IF, et al. Systematic RNA interference reveals that oncogenic KRAS-driven cancers require TBK1. Nature. 2009;462: 108–112. doi:10.1038/nature08460

5.	Mubeen S, Tom Kodamullil AT, Hofmann-Apitius M, Domingo-Fernández D. On the influence of several factors on pathway enrichment analysis. Briefings in Bioinformatics. Oxford University Press; 2022. doi:10.1093/bib/bbac143

Major.8: Results from Cox proportional hazards models are given without the ability to check the assumptions. Making statistics easy does not make them correct.

Response:

Thank you for making such a valuable suggestion. Including additional test statistics in Cox proportional risk model analyses does allow for a more complete assessment of the quality and applicability of the model. Therefore, we added the Schoenfeld residual test to the Cox continuous variable analysis by using the cox.zph() function from the survival package. We add this in the corresponding Methodology section of the manuscript (Page 12, lines 233-236).

Major.9: In the app, for many gene sets, there are unreasonable HRs. For example, for C2CP - Biocarta Bad Pathway, in AML, with the IlluminaHiSeq platform, the HR is 8.16e+04 on the continuous Cox model. This is despite the fact that it is only 1.43 with the median cut-off. I suspect this is due to an issue with pre-processing the data, but there are other possibilities.

Response:

Thank you for your comment. We would like to explain your question as follows: 1) The statistical methods are different. continuous Cox uses Cox one-way regression analysis, while K-M median cut-off performs Kaplan-Meier survival analysis. 2) The subgrouping methods are also different. continuous Cox treats survival time as a continuous variable, while K-M median cut-off takes the median survival time as the cut-off value for binary analysis. Taken together, the two HR values are not comparable. In addition, it is worth noting that the analysis results of continuous Cox and K-M median cut-off simultaneously showed no statistical significance. This reflects the mutual support of the findings of the two statistical methods.

Reviewer #3 Benefits of Using PESSA
Gene Set Activation Levels
The authors argue that PESSA is a tool that distinguishes itself by focusing on gene set activation levels, offering a holistic view of pathway activities rather than individual gene expression levels. This approach can potentially provide more comprehensive insights into the functional status of biological pathways in the context of cancer.

The authors also state that PESSA shows robustness in pan-cancer analysis suggesting its effectiveness in handling diverse cancer types. The main feature is to analyze and compare survival outcomes across different cancer types, potentially revealing common or distinct pathway signatures.

Overall Considerations
PESSA may be particularly valuable for researchers interested in pathway-centric survival analysis across multiple cancer types, but KM Plotter, GEPIA, Prognoscan, and UCSC Xena offer a more extensive range of analyses, making them suitable for diverse research questions. The authors should be able to prove the need for a tool like PESSA and the benefit their tool provided against the specific functionalities and performances offered by each of the abovementioned tools KM Plotter, GEPIA, Prognoscan, and UCSC Xena.

In conclusion, the choice of genomic analysis tools depends on the specific goals and nature of the analyses researchers intend to conduct. PESSA's strengths in gene set activation and pancancer analysis are noteworthy, but authors must carefully consider the trade-offs in comparison to the broader functionalities offered by KM Plotter, GEPIA, Prognoscan, and UCSC Xena.

Response:

Thank you for your detailed and thorough review of our article and for making such valuable suggestions. We have supplemented the table of cross-sectional comparisons of tools in Supplementary table4 with additional notes in the Discussion section of the article (Page 17, lines 324-325). Overall, PESSA is significantly richer than most commonly used tools in terms of the amount of data included, tumor type, type of survival time, and method of survival analysis. In addition, PESSA innovatively uses gene sets based on ssGSEA scores as targets for survival analysis, breaking the previous approach of using only transcriptome expression. Undeniably, PESSA has not yet been able to satisfy users for subgroup analysis, and the sets of marker genes related to each tumor have also been summarized and organized for grouping.
